# Evaluation on the psychological adjustment and countermeasures of civil servants in public emergencies

**DOI:** 10.3389/fpubh.2022.1114518

**Published:** 2023-01-20

**Authors:** Tianyi Zhu, Shijie Guo, Wenfeng Li, Zhuang Li

**Affiliations:** ^1^College of Political Science and Law, Jiangxi Normal University, Nanchang, Jiangxi, China; ^2^School of Marxism, Ocean University of China, Qingdao, Shandong, China

**Keywords:** civil servants' psychological adjustment, public emergencies, public health, psychological response measures, comprehensive psychological quality

## Abstract

Public health emergencies are inevitable major development crises, and there are almost no omens of any emergency. The current social development would inevitably affect the psychological situation of civil servants. Grass roots civil servants have a wider range of tasks, more difficult working conditions and a more difficult environment. Under the strong social pressure, civil servants would also have negative factors such as fear and negative attitude. The mental health of grass-roots civil servants depends not only on the image and efficiency of the government, but also on creating a harmonious atmosphere and the quality of economic development. Therefore, people must pay attention to the psychological health of civil servants. It is mainly through psychological intervention and psychological adjustment to improve mental health. By analyzing the psychological characteristics of civil servants under emergencies and under pressure, and according to the importance of their coping ability under emergencies, this paper conducted corresponding psychological adjustment and psychological intervention to ensure the psychological health of civil servants, improve their ability to deal with public emergencies, and enable them to use correct and positive psychology to deal with public emergencies. It can be seen from the firefly algorithm that the prediction error value of the comprehensive quality of civil servants was declining, while the evaluation effect of the comprehensive quality was rising. The average value of the prediction error value of the comprehensive quality was about 0.49, and the average value of the evaluation effect of the comprehensive quality was about 0.73. In the whole process, the prediction error value of comprehensive quality decreased by 0.37, and the evaluation effect of comprehensive quality increased by 0.33. The comprehensive psychological quality and psychological adjustment ability of civil servants after psychological intervention were better than those before psychological intervention. The comprehensive psychological quality of civil servants after psychological intervention was 8.56% higher than that before psychological intervention, and the psychological adjustment ability was 8.47% higher than that before psychological intervention.

## 1. Introduction

Public health emergencies show that governance is the most effective means to stabilize public order. When life is threatened, the ability of government officials to respond to emergencies and the impact of their ability to respond is a major challenge for government officials, because the handling effect of emergencies affects the public's attitude toward the government. In this regard, this paper analyzes the impact of civil servants' emergency response capacity on health emergencies, reviews the psychological situation of civil servants in health emergencies, and puts forward appropriate plans to improve their psychological situation, which has important practical significance for the psychological adjustment of civil servants.

The psychology of civil servants is a research that cannot be ignored. Borst et al. (1) used the insights in the public management literature to expand the job search demand resource model of work participation, and public organizations can choose people with positive personality and high public service motivation by adding work related resources, improving the speed of emergency response by selecting high-level managers. Borst and Lako (2) combined the work demand resource model and high-performance work practice classification, and analyzed the determinants of pride, The professional pride of civil servants would hardly be affected by high performance work practices. Costantini (3) studied the extent to which the improvement of psychological capital as a personal resource can improve the work participation of public sector employees, aiming to investigate the intervention measures to improve the work participation by enhancing psychological capital. Qing (4) investigated the impact of moral leadership on employee attitudes (emotional commitment and job satisfaction), and studied the role of psychological empowerment as a potential mediator of these relationships. Borst (5) found out whether the impact of work participation on the attitude, behavior and performance results of the non-public sector and the public sector was as high as expected, and whether these relationships were different among the public, non-public and private sectors. Husain (6) explored the obstacles preventing Pakistanis from seeking psychological help, and suggested that mental health practitioners in the country improve their awareness of mental health and improve mental health services. Duran et al. (7) believed that psychological contract can provide rich insights for understanding the relationship between employees and employers within the police, as well as the pressure and wellbeing of the police, and used framework analysis to analyze these interviews. The above studies all described the psychological characteristics of civil servants, but did not describe psychological intervention measures.

Many scholars have studied the psychological intervention measures of civil servants. Wang believed that while controlling the epidemic, the government should also pay attention to the mental health of the general public, medical workers and patients with mental disorders. Other measures for patients with mental disorders may play a crucial role during the pandemic (8). Saito (9) estimated the changes in mental health, quality of life and unemployment experience of ordinary workers during the first COVID-19 outbreak in Japan. The purpose is to assess the psychological changes and impact of public servants in emergency. Zou (10) found that public trust in the government is conducive to alleviating their psychological crisis through regression analysis of public questionnaire data. Zhong (11) investigated the psychological status and countermeasures of hospital guidance nurses, and analyzed the interview data with color analysis method to provide strong psychological support for the triage work of nurses. The above studies all described the measures of psychological intervention, but there are still some deficiencies in psychological adjustment.

At present, there are few researches on the psychological health of civil servants, which are all individual studies, with no common analysis and problems such as standardization, decentralization and alienation. The neglect of civil servants' mental health not only has a negative impact on the development of civil servants themselves, but also has a negative impact on the improvement of the political system and the healthy development of society. Mental ill health would affect social governance and emergency handling. The mental health problem of civil servants has destroyed the ecological balance between the administrative system and the administrative structure, and reduced the attitude and efficiency of civil servants at the grass-roots level, which has seriously affected the efficiency of social services and public affairs management, and hindered the exercise of administrative power.

## 2. Evaluation of the psychological status of civil servants in public emergencies

### 2.1. Characteristics of public emergencies

**Figure 1:**
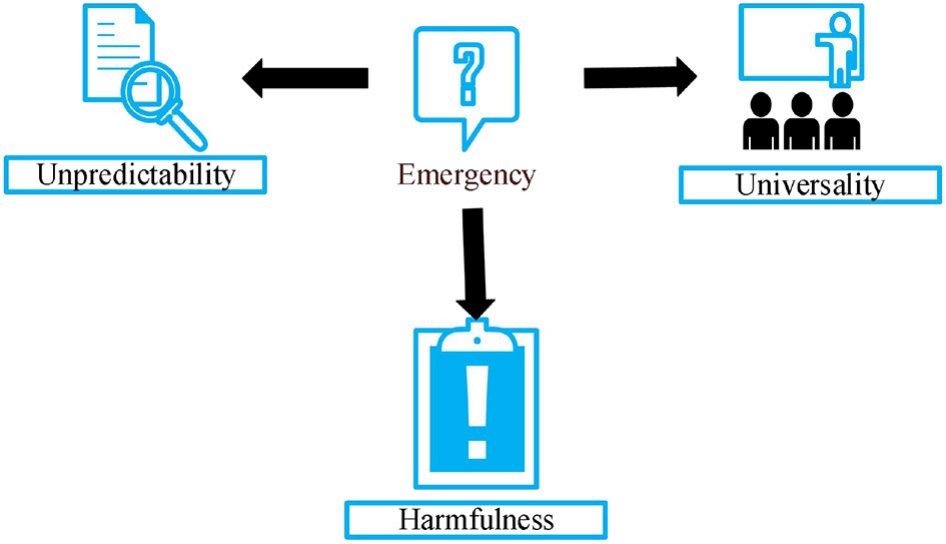
Characteristics of public emergencies.

There are three main characteristics of public emergencies, as shown in Figure 1. First, it is very unpredictable. An emergency is a temporary emergency that does not occur in the normal process of development, nor is it consistent with the normal trend of development. The operation of modern society often has certain regularity, and many social problems must be standardized, mainly by improving the psychological quality of government administrators. However, due to some subjective factors, the laws of social development and working rules would be broken, which makes unforeseen events different from the development trend and normal operating procedures. Because of the variety of factors that cause emergencies, it is difficult to predict in advance, which is the unpredictability of emergencies. Second, it is harmful to society. In general, emergencies often have a negative impact on social and economic development and the personal and financial security of citizens. For example, it is difficult to prevent and predict public security events that are centrally handled in social emergencies in advance, and most of the fund time would involve personal and property safety, which is extremely harmful to society (12). The third is the universality of social consequences. With the rapid development of society, the relationship between all social strata and citizens is increasingly close. In this regard, the social impact of emergencies would continue to expand, and the scope of their impact would continue to expand. Sometimes, regional emergencies worsen in a short time, leading to a chain reaction. In this sense, if the public administration department cannot respond to the emergency quickly and effectively, its negative impact would continue to increase, causing more serious and deeper losses.

### 2.2. Psychological characteristics of civil servants

In emergencies, the psychological characteristics of civil servants mainly include the following. The first is physical symptoms. In an emergency, most civil servants suffer from insomnia and are weak. These physical symptoms often reflect unhealthy psychology that brings pain to civil servants. In addition, unhealthy lifestyles and habits have increased the burden on civil servants. The second is the imbalance of desire. In an emergency, civil servants often excessively pursue their own interests and needs, lack a sense of social morality, and lack a sense of security. They alsp have a strong sense of inferiority in psychology, cannot correctly treat the dignity of others, and use sarcastic language to describe the achievements of others. Although some civil servants have low positions and limited power, the wage gap between department employees is determined by increasing employment opportunities and expanding interpersonal relationships, resulting in some civil servants' psychological imbalance and desire for material benefits (13). The third is job weariness. Because of the complexity of work and emergency situations, civil servants often feel depressed and negative. Their interest and enthusiasm in work have decreased, and their activity and creativity have decreased. Their attention and memory have weakened, and their spirit and working state are also poor. When encountering difficulties and setbacks in work, he is afraid of difficulties and dangers and indifferent to work, mainly by improving the psychological quality of government administrators. The fourth is fear. The overall quality of civil servants has been continuously improved, but the competition for emergency response has become increasingly fierce. The fierce competition has aroused the anger, anxiety and hesitation of officials. Moderate anxiety can improve the morale of civil servants, but excessive anxiety would affect their normal work and life, cannot handle state affairs satisfactorily, cannot tolerate the masses, or even cannot argue with the masses, which leaves a bad impression on the masses.

### 2.3. The dual role of the psychological pressure of civil servants

**Figure 2:**
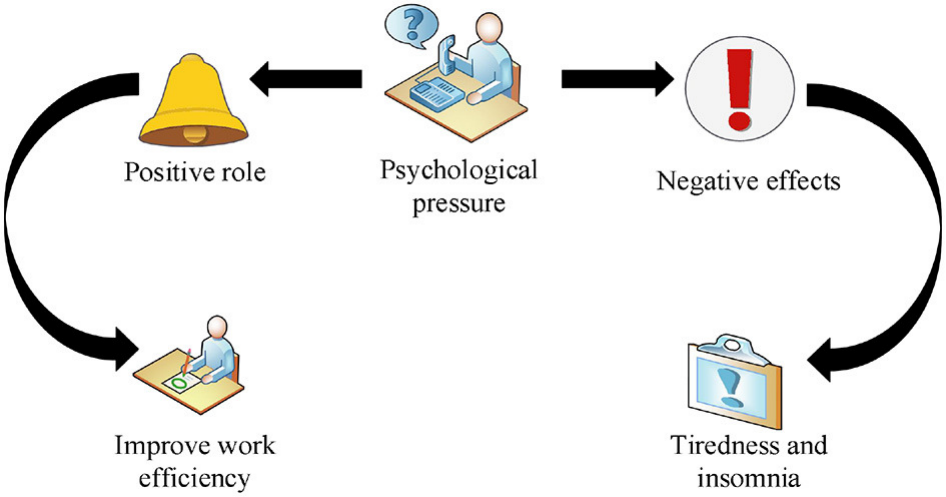
The dual role of civil servants' psychological pressure.

The psychological pressure generated by civil servants in emergencies has both positive and negative effects, as shown in Figure 2. The first is the positive effect. Under the maximum pressure, the range of high-performance work performance is the best pressure zone. In this regard, work must be carried out under personal pressure, while the business value in this area is relatively high. From an individual perspective, in areas where work pressure is more appropriate, people can exercise individual psychological endurance and encourage them to actively adjust their mentality (14). In the best pressure zone, civil servants work more actively. When they encounter problems in life and work, they would become more calm. From the perspective of organizational efficiency, if the workload is placed in a more appropriate environment, it can improve efficiency. The second is the negative impact. The pressure on employees exceeds the optimal pressure, which greatly reduces the work efficiency of employees. In particular, the workload is too small, and the working conditions are too good. It is difficult to mobilize the enthusiasm of civil servants, resulting in a significant reduction in the enthusiasm and concentration of staff, which greatly reduces the efficiency of civil servants. If the pressure is high, officials may lose sleep. When the pressure exceeds the critical point, civil servants would feel tired.

## 3. Psychological adjustment and intervention measures of civil servants in public emergencies

### 3.1. The necessity of psychological adjustment of civil servants in emergencies

First, the development trend of public emergencies has brought new challenges. With the destruction of the natural environment, the acceleration of population and social mobility, the deepening of the market mechanism and the transformation of societies in various countries, the occurrence of natural disasters has not only increased, but also the number of social emergencies in the political, economic and social fields has increased, making these problems more and more difficult to solve. These unforeseeable social events not only pose a serious threat to people's lives, but also cause great damage to social economic development and social stability due to the outbreak of social events, because the deterioration of social events would affect the public's sense of trust in the government. At present, the main trend of events related to unforeseen social events is that the number of such events is increasing, and their impact is expanding. The frequency, scale and risk of unexpected social events are also increasing. It rapidly spreads the information of public emergencies, and expands the scope and increases the fluctuation frequency. It can also be seen that changes in emergencies seem to pose new challenges to the ability of civil servants to cope with emergencies. Second, emergency relief is an important task for civil servants who are responsible for responding to emergencies. Social crisis management is a dynamic process. Civil servants are the main body of national crisis management, controlling this dynamic process and managing the entire management process. Therefore, the crisis management ability of civil servants plays an important role in managing public crises. Civil servants usually assume corresponding political, management and work responsibilities and represent the image of the government or departments to some extent. They play an important role in emergency management. Civil servants are the focal points, advocates and executors of the government and senior management, as well as lawyers, decision-makers and crisis observers at the local level (15). The crisis is characterized by suddenness and universality, which puts forward higher requirements for the emergency response ability of civil servants. Timely and appropriately solving the national crisis that would undermine stability and unity can not only solve the problems that affect the daily life of citizens, but also bring much convenience to society and people. Therefore, the ability of civil servants to deal with emergencies must be strengthened.

### 3.2. Problems of civil servants in dealing with emergencies

**Figure 3:**
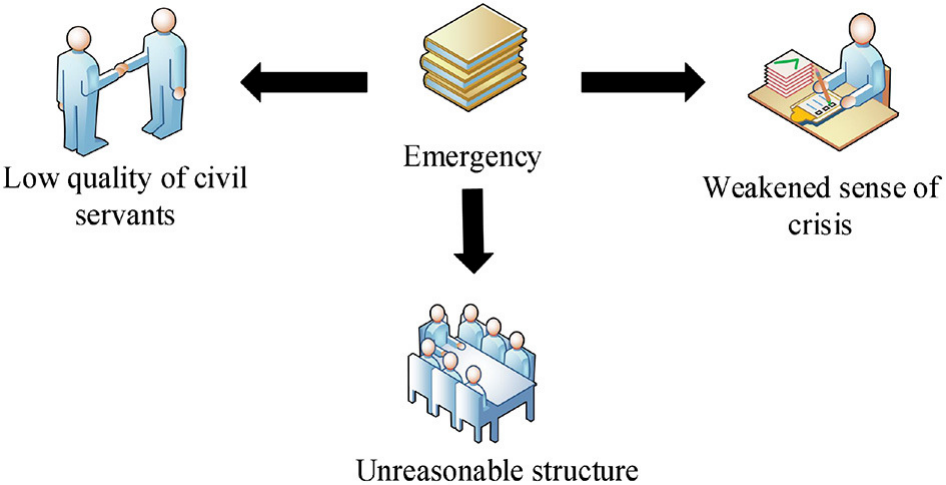
Problems exposed by civil servants in response to emergencies.

There are mainly three kinds of problems exposed by civil servants when dealing with emergencies, as shown in Figure 3. First, the quality of civil servants is low. In general, the qualifications and qualities of civil servants meet the needs of public administration. However, in the emergency crisis, the mentality and behavior of civil servants are still insufficient, which indicates that they have some problems in coping with the crisis, mainly the confusion and psychological escape when dealing with emergencies. Second, the structure of the organization is unreasonable. The current national crisis management system mainly depends on the existing administrative power of governments at all levels, which affects the effectiveness of crisis management. The emergency preparedness is incomplete and the emergency work is not carried out. The administrative functions of the government are unbalanced, and the single emergency function and information communication are not timely. In emergencies, communication is an important task, and the role of non-governmental organizations has been weakened. In an emergency, society is not the object of passive mobilization. Mature societies can also mobilize actively in crisis situations, even before the government. Third, the sense of crisis has weakened. The quality of some civil servants does not meet the actual needs. The most serious defect is the lack of awareness of the seriousness of social emergencies and the importance of crisis management, and the lack of understanding of the crisis management and management rules of social emergencies. Therefore, their awareness of ideological crisis is weak. They lack effective measures to prevent and deal with social emergencies stipulated by law, and their awareness and quality of dealing with emergencies are very poor.

### 3.3. Psychological intervention measures for civil servants in emergencies

**Figure 4:**
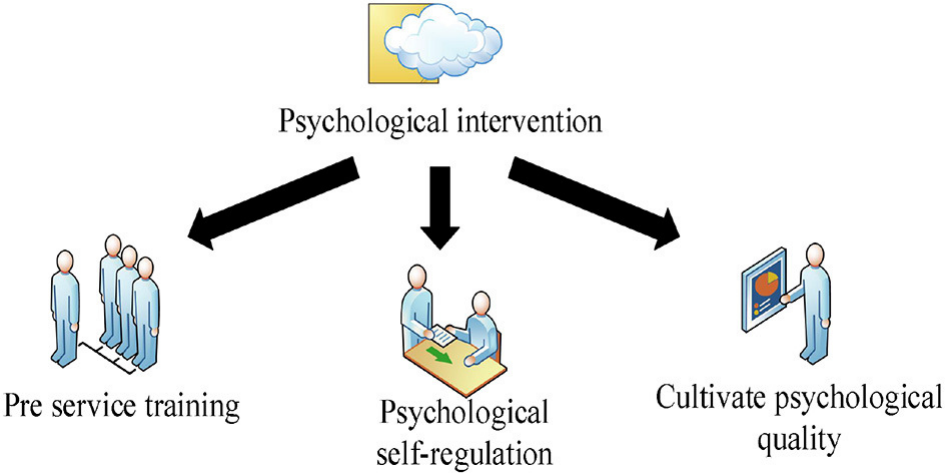
Psychological intervention of civil servants in emergencies.

The psychological intervention of civil servants in emergencies can be started from the following aspects, as shown in Figure 4.

(1) Pre-service training.

People need to guide civil servants to take up their posts, so that they have certain psychological expectations. In addition, a problem reporting center would be set up within the unit to provide information and advice to civil servants on work, family or psychological problems, actively promote and support the resolution of these psychological problems, and protect their privacy and sense of security. Most of them are biased against users of consulting services, which means that civil servants cannot receive timely medical care when mental health problems occur, and they lose confidence in senior managers and their stable work. To this end, the government and enterprises should regularly carry out psychological literacy activities so that civil servants can collectively solve psychological problems, mainly to improve the psychological endurance to deal with emergencies. In addition, the psychological problems of civil servants should not be disadvantaged, excluded from society or discriminated against. On the contrary, these problems need more care, help and warmth. In addition, in the work and daily life of civil servants, people must give them some positive evaluation and encouragement, promote mutual support and coordination between civil servants, make them closer to the psychological quality standards of civil servants, and expand their communication opportunities. This would not only improve their work efficiency, but also ensure a more harmonious interpersonal relationship between civil servants.

(2) Psychological self-regulation.

The first is to master the adaptability. If people decide to work in the civil service, people should be prepared mentally. The public sector faces many challenges, pressures and setbacks. In the face of failure, civil servants must overcome failure and recognize the nature of failure. Everyone would fail, and failure would not last forever. The psychological performance of civil servants would not continue to be weak after they are used to failure. If people feel depressed and anxious, people should avoid overloading, set priorities and the right date, leave room for maneuver, and find ways to relax. In addition, increasing physical activity can alleviate the physical symptoms of diseases and reduce the occurrence of psychological problems.

(3) Cultivating psychological quality.

First of all, people must provide high-quality psychological education for the government, society and individuals. In order to improve the psychological level of civil servants, social training institutions should look for psychologists and offer psychological training courses to cultivate the psychology of civil servants. Secondly, it is the positive influence of like-minded people, social environment and cultural media. The third is to help civil servants enhance their sense of identity and better serve the people by cultivating their psychological quality. Finally, people must consciously combine learning, career and personal life to develop strengths and eliminate weaknesses. People must further strengthen the psychological identity of civil servants, strengthen mutual trust and interaction between civil servants, and strengthen the unity and cooperation within the team through high-quality training for different groups.

## 4. Application of firefly algorithm in the psychological adjustment of civil servants

In order to study the psychological status of civil servants under emergencies and the specific effect of adjustment and response, this paper analyzes the psychological experience effect and actual psychological score of civil servants under emergencies through the firefly algorithm, and selects corresponding adjustment measures according to the scoring indicators, so as to improve the psychological quality of civil servants. Firefly algorithm calculates comprehensive evaluation index and prediction error through fitness function. First of all, this paper uses the firefly algorithm to propose a strategy to help civil servants improve their psychological quality:


(1) $$\begin{array}{lll} A_{i} & = & A_{i}+\alpha \left(x\right)\times \left(A_{j}-A_{i}\right)+\beta \times \delta_{i} \end{array}$$

(2) $$\begin{array}{lll} \alpha \left(x\right) & = & \alpha_{0}e^{xij} \end{array}$$

Among them, Formula (2) refers to the attraction of high psychological quality to low psychological quality, and β, δ_*i*_ refers to the step size factor and random factor of improving psychological quality strategy. This paper then analyzes the adaptability function of civil servants' psychology under emergencies as follows:


(3) $$\begin{array}{l} \min y\left(S_{i},T_{i}\right)=\sqrt{\frac{1}{m}\overset{m}{\underset{i=1}{\sum }}{\left(a\left(i\right)-â\left(i\right)\right)}^{2}} \end{array}$$

*S*_*i*_, *T*_*i*_ refers to the strategy of improving psychological quality and the effect of improvement, and *m* refers to the training strategy of psychological adjustment. Then it analyzes the evaluation effect and relevant evaluation indicators of the comprehensive quality of civil servants' psychological adjustment under emergencies:


(4) $$\begin{array}{l} Z=\sqrt{\frac{1}{m}\overset{m}{\underset{n=1}{\sum }}{\left(a_{n}-{â}_{n}\right)}^{2}} \end{array}$$

(5) $$\begin{array}{l} Q=\frac{\overset{m}{\underset{n=1}{\sum }}a_{n}{â}_{n}}{\sqrt{\overset{m}{\underset{n=1}{\sum }}a_{n}^{2}}\sqrt{\overset{m}{\underset{n=1}{\sum }}{â}_{n}^{2}}} \end{array}$$

Then, according to the comprehensive evaluation effect and relevant evaluation indicators, the final prediction error value of civil servants' comprehensive psychological quality is:


(6) $$\begin{array}{l} R=Z\cdot \frac{\min y\left(a_{n}{â}_{n}\right)}{Q} \end{array}$$

## 5. Experimental evaluation of the psychological adjustment of civil servants in emergencies

In order to study the psychological changes and self-regulation of civil servants in emergencies, this paper analyzed the predictive value of the comprehensive psychological quality of civil servants' psychological adjustment and the comprehensive evaluation effect through the firefly algorithm, and made the corresponding psychological adjustment plan according to the comprehensive evaluation, so as to improve the psychological quality of civil servants. First of all, this paper investigated the satisfaction of civil servants in three regions with the psychological intervention strategies proposed in the paper, including 100 civil servants in each region, as shown in Table 1.

**Table 1:** Satisfaction of civil servants in three regions with psychological intervention strategies.

	**Satisfied**	**Commonly**	**Dissatisfied**
Region 1	87	7	6
Region 2	85	6	9
Region 3	86	9	5
Total	258	22	20

**Figure 5:**
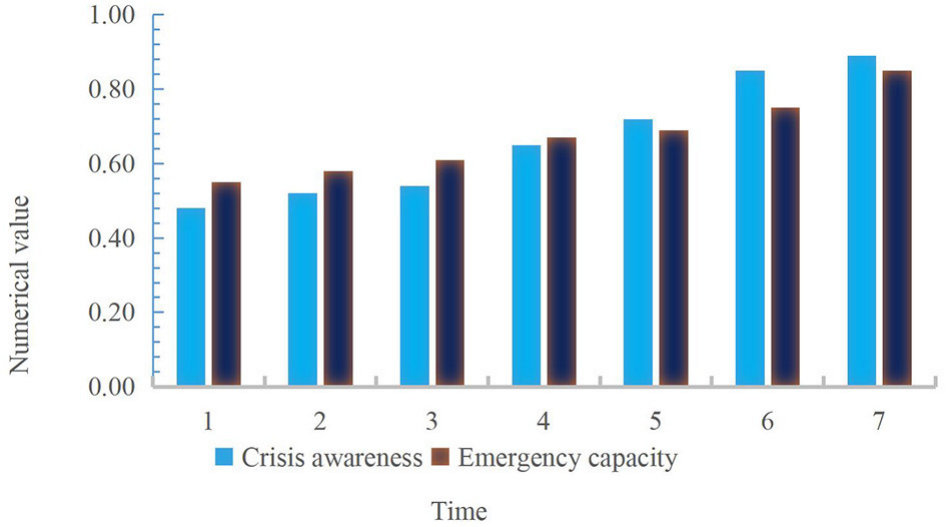
Civil servants' crisis awareness and emergency response ability under the implementation of psychological strategies.

According to the data described in Table 1, civil servants in the three regions were generally satisfied with the psychological intervention strategies proposed in the paper. Among the satisfied civil servants, Region 1 had the most satisfied civil servants, accounting for 33.7% of the total, and Region 2 had the least satisfied civil servants, accounting for 32.9% of the total. Among the general civil servants, the number of civil servants in Region 3 was the largest, accounting for 40.9% of the total, while the number of civil servants in Region 2 was the smallest, accounting for 27.3% of the total. Among the unsatisfied civil servants, the number of civil servants in Region 2 was the largest, accounting for 45% of the total number of unsatisfied civil servants, while the number of civil servants in Region 3 was the smallest, accounting for 25% of the total number of unsatisfied civil servants. On the whole, 86% of the total number of civil servants in the three regions were satisfied; 7.3% were generally satisfied; 6.7% were dissatisfied. Satisfied civil servants believed that the psychological intervention strategy can reduce the psychological infection of emergencies to them, and avoid the anxiety caused by their inability to deal with emergencies in time or well. The dissatisfied civil servants thought that the psychological intervention strategy did not help to improve the psychological quality, because they think their psychological endurance is no problem, and psychological treatment would affect their reputation. This paper then analyzed the crisis awareness and emergency response ability of civil servants under the implementation of psychological strategies. The specific changes of the two are shown in Figure 5.

**Figure 6:**
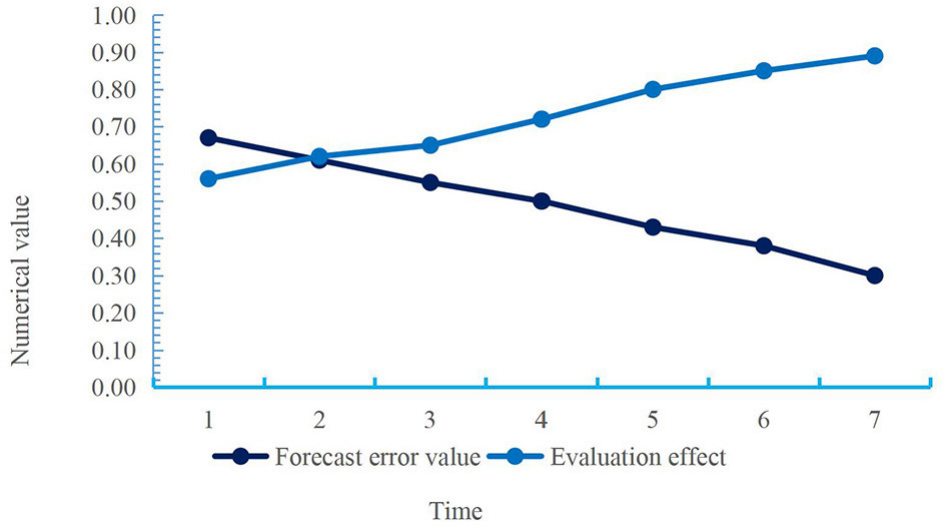
Prediction error value and evaluation effect of civil servants' comprehensive quality.

According to the data described in Figure 5, under the implementation of psychological strategies, the crisis awareness and emergency response capacity of civil servants have gradually increased over time, with the average value of crisis awareness of civil servants being about 0.66 and the average value of emergency response capacity being about 0.67. On the whole, the initial value of civil servants' crisis awareness was 0.48, which increased to 0.89 on the seventh day, an increase of 0.41 in the whole process; the initial emergency capacity of civil servants was 0.55, which increased to 0.85 on the seventh day, an increase of 0.30 in the whole process. The increase of crisis awareness and emergency response ability of civil servants under the psychological intervention strategy showed that strong psychological quality can help civil servants improve their comprehensive quality and ability to cope with difficulties, and avoid negative emotions such as anxiety due to inability to solve problems. It not only improved the psychological endurance, but also improved the ability to solve emergencies. Then the firefly algorithm was used to analyze the prediction error value of the comprehensive quality of civil servants and the evaluation effect. The specific changes are shown in Figure 6.

**Figure 7:**
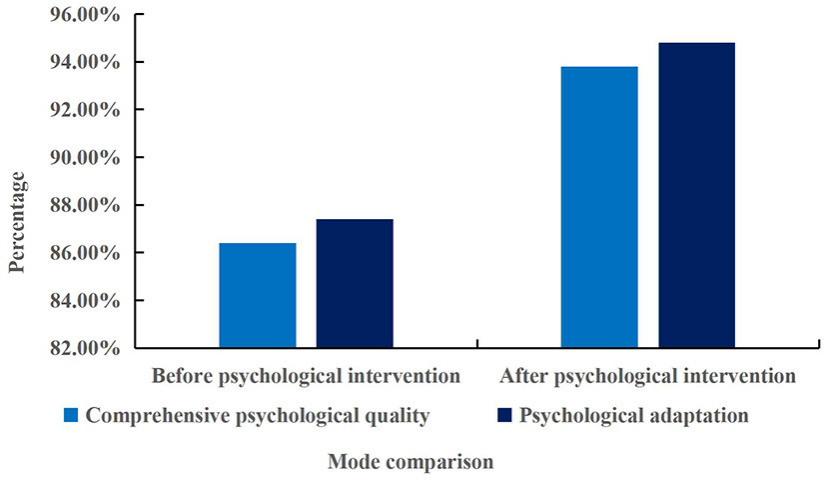
Comprehensive psychological quality and psychological adjustment ability of civil servants after psychological intervention.

According to the curve depicted in Figure 6, the prediction error value of civil servants' comprehensive quality was declining, while the evaluation effect of comprehensive quality was rising. The average value of the prediction error value of comprehensive quality was about 0.49, and the average value of the evaluation effect of comprehensive quality was about 0.73. In the whole process, the prediction error value of comprehensive quality decreased by 0.37, and the evaluation effect of comprehensive quality increased by 0.33. The decline of the prediction error value of comprehensive quality showed that the psychological detection of civil servants was more accurate after psychological intervention, which was convenient to guide their psychological development in a timely manner, mainly improving their psychological quality, and laying a foundation for the handling of future emergencies and social governance. The increase in the evaluation effect of comprehensive quality showed that the overall quality and ability of civil servants to deal with emergencies have become stronger and stronger after psychological intervention. Finally, the comprehensive psychological quality and psychological adjustment ability of civil servants after psychological intervention were analyzed and compared with the data before psychological intervention, as shown in Figure 7.

According to the comparison chart in Figure 7, the comprehensive psychological quality and psychological adjustment ability of civil servants after psychological intervention were better than those before psychological intervention. The comprehensive psychological quality of civil servants after psychological intervention was 8.56% higher than that before psychological intervention, and the psychological adjustment ability was 8.47% higher than that before psychological intervention. The implementation of psychological intervention strategy can improve the psychological endurance of civil servants, and make them always maintain a positive and objective attitude to face various difficulties, and would not give up because of setbacks. Through the psychological intervention strategy, the adaptability and psychological status of civil servants were better than before the psychological intervention, and the overall quality of civil servants has also improved a lot.

## 6. Conclusions

Public emergencies not only contribute to the psychological development and psychosocial optimization of civil servants, but also have a negative impact on their mental health and psychosocial optimization. Today's social reality is like a double-edged sword. On the one hand, it provided more opportunities for civil servants to realize themselves and develop their potential, and promoted their mental health and sound social thinking; on the other hand, the rapid changes in society have increased the psychological pressure of civil servants, posed an actual or potential threat to their mental health, and caused many problems to their psychosocial situation, thus making it more difficult for civil servants to adapt. This is a greater demand for the psychological skills of civil servants and a major challenge to the provision of psychosocial services. The analysis of the psychological problems faced by civil servants provides information about psychological intervention and lays a foundation for strengthening the emergency response capacity of civil servants.

## Data availability statement

The original contributions presented in the study are included in the article/supplementary material, further inquiries can be directed to the corresponding author.

## Author contributions

All authors listed have made a substantial, direct, and intellectual contribution to the work and approved it for publication.

## References

[1] Borst RT Kruyen PM Lako CJ. Exploring the job demands–resources model of work engagement in government: bringing in a psychological perspective. Rev Public Person Admin. (2019) 39:372–97. 10.1177/0734371X17729870

[2] Borst RT Lako CJ. Proud to be a public servant? An analysis of the work-related determinants of professional pride among Dutch public servants. Int J Public Admin. (2017) 40:875–87. 10.1080/01900692.2017.1289390

[3] Costantini A. Work engagement and psychological capital in the Italian public administration: a new resource-based intervention programme. SA J Ind Psychol. (2017) 43:1–11. 10.4102/sajip.v43i0.1413

[4] Qing M. Exploring the impact of ethical leadership on job satisfaction and organizational commitment in public sector organizations: the mediating role of psychological empowerment. Rev Manag Sci. (2020) 14:1405–32. 10.1007/s11846-019-00340-9

[5] Borst RT. The attitudinal, behavioral, and performance outcomes of work engagement: a comparative meta-analysis across the public, semipublic, and private sector. Rev Public Person Admin. (2020) 40:613–40. 10.1177/0734371X19840399

[6] Husain W. Barriers in seeking psychological help: public perception in Pakistan. Commun Mental Health J. (2020) 56:75–8. 10.1007/s10597-019-00464-y 31542848

[7] Duran F Woodhams J Bishopp D. An interview study of the experiences of police officers in regard to psychological contract and wellbeing. J Police Crim Psychol. (2019) 34:184–98. 10.1007/s11896-018-9275-z

[8] Wang S. Psychological influence of coronovirus disease 2019 (COVID-19) pandemic on the general public, medical workers , and patients with mental disorders and its countermeasures. Psychosomatics. (2020) 61:616–24. 10.1016/j.psym.2020.05.005 32739051 PMC7255244

[9] Saito S. Psychological impact of the state of emergency over COVID-19 for non-permanent workers: a Nationwide follow-up study in Japan. BMC Public Health. (2021) 21:1–12. 10.1186/s12889-021-10401-y 33573632 PMC7877331

[10] Zou D. Psychological factors and moral intervention countermeasures of COVID-19 later stage government overall management to the public under the network information environment. Tobacco Regul Sci. (2021) 7:6735–48. 10.18001/TRS.7.6.1.06

[11] Zhong M. Investigation and countermeasures of psychological status of outpatient guide nurses during the outbreak of COVID-19 pandemic. Am J Nurs. (2020) 9:191–4. 10.11648/j.ajns.20200904.11

[12] Li X. Analysis on existing problems and counter measures of public health management in the new era. Sci Soc Res. (2022) 4:38–43. 10.26689/ssr.v4i7.4160

[13] Chen CA Chen DY Xu C. Applying self-determination theory to understand public employee's motivation for a public service career: an East Asian case (Taiwan). Public Perform Manag Rev. (2018) 41:365–89. 10.1080/15309576.2018.1431135

[14] Prysmakova P Vandenabeele W. Enjoying police duties: public service motivation and job satisfaction. J Police Crim Psychol. (2020) 35:304–17. 10.1007/s11896-019-09324-7

[15] Levitats Z Vigoda-Gadot E. Emotionally engaged civil servants: toward a multilevel theory and multisource analysis in public administration. Rev Public Person Admin. (2020) 40:426–46. 10.1177/0734371X18820938

